# Effect of Aging, Antioxidant, and Mono- and Divalent Ions at High Temperature on the Rheology of New Polyacrylamide-Based Co-Polymers

**DOI:** 10.3390/polym9100480

**Published:** 2017-10-04

**Authors:** Saeed Akbari, Syed M. Mahmood, Isa M. Tan, Onn Lin Ling, Hosein Ghaedi

**Affiliations:** 1 Centre of Research in Enhanced Oil Recovery (COREOR), Petroleum Engineering Department, Universiti Teknologi PETRONAS, Seri Iskandar, 32610 Tronoh, Malaysia; 2 Shale Gas Research Group (SGRG), Institute of Hydrocarbon Recovery, Petroleum Engineering Department, Universiti Teknologi PETRONAS, Seri Iskandar, 32610 Tronoh, Malaysia; 3 Fundamental and Applied Science, Universiti Teknologi PETRONAS, Seri Iskandar, 32610 Tronoh, Malaysia; isa.mtan57@gmail.com; 4 Petroleum Engineering Department, Universiti Teknologi PETRONAS, Seri Iskandar, 32610 Tronoh, Malaysia; onnlin.ling@gmail.com; 5 Chemical Engineering Department, Universiti Teknologi PETRONAS, Seri Iskandar, 32610 Tronoh, Malaysia; ghaedi.hosein63@gmail.com

**Keywords:** rheology, AN132 VHM, FLOCOMB, SUPERPUSHER, THERMOASSOCIATIF, thermal stability

## Abstract

The viscosity of four new polymers was investigated for the effect of aging at high temperature, with varying degrees of salinity and hardness. The four sulfonated based polyacrylamide co-polymers were FLOCOMB C7035; AN132 VHM; SUPERPUSHER SAV55; and THERMOASSOCIATIF copolymers. All polymer samples were aged at 80 °C for varying times (from zero to at least 90 days) with and without isobutyl alcohol (IBA) as an antioxidant. To see the effect of divalent ions on the polymer solution viscosity, parallel experiments were performed in a mixture of CaCl_2_-NaCl of the same ionic strength as 5 wt % NaCl. The polymers without IBA showed severe viscosity reduction after aging for 90 days in both types of preparation (5 wt % NaCl or CaCl_2_-NaCl). In the presence of IBA, viscosity was increased when aging time was increased for 5 wt % NaCl. In CaCl_2_-NaCl, on the other hand, a viscosity reduction was observed as aging time was increased. This behavior was observed for all polymers except AN132 VHM.

## 1. Introduction

Water flooding has been used as an improved oil recovery technique for decades in order to displace oil and to maintain reservoir pressure [1]. The viscosity of water is often lower than the viscosity of oil, which causes water to move faster than oil. This makes the water displacement front unstable [2]. The addition of polymer to the injected water can greatly tackle this problem by increasing the viscosity of the injected water, thus a mobility control between the water and the hydrocarbons will be provided and sweep efficiency will be increased [3,4,5].

Unhydrolyzed polyacrylamide-based polymer was one of the main synthetic polymers initially considered by the oil industry in the polymer flooding process because of its chemical stability. However, its ability to increase viscosity was low and they encountered high adsorption onto mineral surfaces causing significant loss [5,6]. 

**Figure 1:**
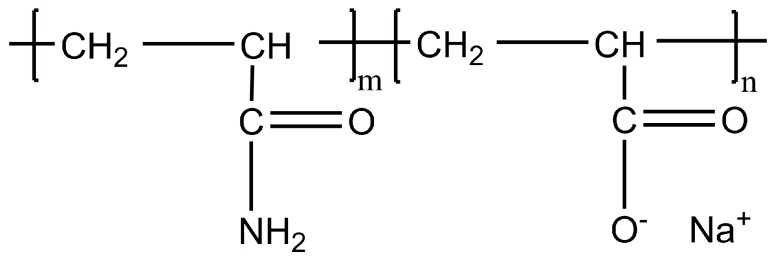
Copolymer of acrylamide and sodium acrylate (HPAM).

As an improvement, partially hydrolyzed polyacrylamide (HPAM) was suggested, by converting some of the amide group on the polyacrylamide (PAM) to the carboxylate group during the hydrolysis process [5], see Figure 1. Electrostatic repulsion caused by negative charges of the carboxylate groups distributed along the polymer chain helps the polymer to be uncoiled easily and therefore provides higher viscosity even at low polymer concentrations [7,8]. In addition, polymer losses due to adsorption on the rock surfaces in porous media is reduced because both the carboxyl group of HPAM and sandstone surfaces are negatively charged and repel each other [3,9,10].

Although HPAM is an economically and technically good candidate for application in mild conditions, it has serious limitations for use in harsh reservoir conditions. Thermal degradation and high adsorption are encountered when the temperature is higher than 70 °C, the formation water has a TDS (total dissolved solids) of more than 40,000 ppm, and it contains divalent ions [11,12,13,14]. 

High reservoir temperatures result in hydrolysis of more amide groups, which is unfavorable after a certain limit. By passing this limit, and if the brine contains significant amounts of divalent cations such as calcium and magnesium, a viscosity drop will occur due to ionic bridges that can ultimately result in precipitation [12].

There is a need to enlarge the application envelope of polymer flooding to high-temperature and high-salinity conditions. Either classical polymers should be modified or new polymers should be developed which can resist hydrolysis in high temperature and provide a thermal and chemical stable polymer for Enhanced Oil Recovery (EOR) applications [6]). The newly modified polymers are mainly HPAM-based co-polymers functionalized with 2-Acrylamido-2-Methylpropane Sulfonate (AMPS) monomers [15,16]. Table 1 lists four of these modified polymers that were investigated in this research. 

Since polymer flooding is a long-term process lasting for several months to years, the degradation of polymers (resulting in viscosity loss) with time is a major concern, especially in high-temperature and high-salinity oil reservoirs. A good polymer candidate for EOR should not only have suitable viscosity and good compatibility with reservoir brine, but also maintain thermal stability over time. The remaining viscosity after aging is one of the primary criteria for any polymer to be used in EOR [17,18]. Therefore, it is important for the design of a successful polymer flood that the thermal stability of polymer be investigated in the laboratory under reservoir conditions.

Since the first publications on these modified polymers appeared in 2015 and later (FLOCOMB in 2016) [19,20,21], and they have recently emerged, their rheological behavior as influenced by aging, salinity, hardness, and oxidation has not been thoroughly studied and published. The primary target of this study was to fill this gap. To study the effect of aging, two sets of polymer samples were prepared, one with 5 wt % NaCl and the other with a mixture of NaCl and CaCl_2_, such that the ionic strength of both solvents was equal. Since four polymers were studied, there were eight bulk solutions prepared altogether. From these bulk solutions, small samples of 10 mL were taken out and aged mainly for 0, 1, 7, 90 days at 80 °C. After aging at high temperature was complete, these samples were tested for apparent viscosity in aerobic conditions at 25 °C. In order to understand the effect of oxidation on rheological behavior, the above series of experiments were repeated under anaerobic conditions by adding antioxidants as soon as the solution was prepared and before aging began.

## 2. Materials and Methods 

### 2.1. Polymers Descriptions

Four co-polymers were selected for this experiment. A brief description of each follows:

FLOCOMB C7035: This polymer is an anioic post-HPAM [22]. Through a post-hydrolysis process, molecular weight and polydispersity (i.e., molecular weight distribution) were increased so as to provide a full range of tolerant calcium showing chemical stability in very hard and salty brines [19,23].

**Figure 2:**
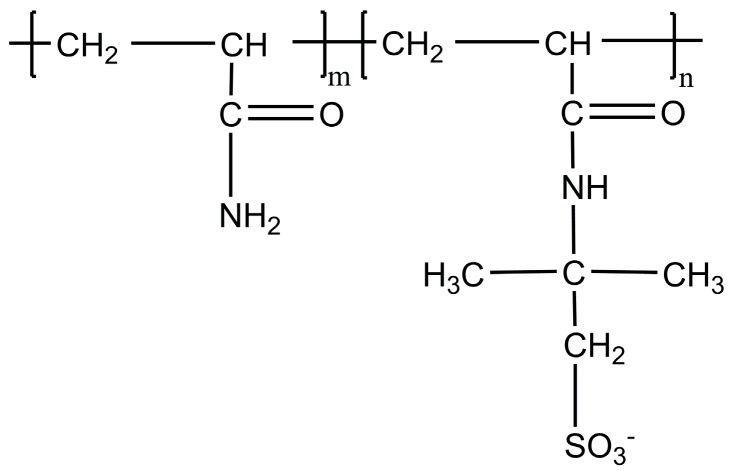
Molecular structure of sulfonated polyacrylamide polymers.

AN132 VHM: A sulfonated polyacrylamide copolymer of acrylamide (AM) with AMPS with a 32 mol % sulfonation degree and high molecular weight [21,23,24], see Figure 2. Such a polymer, with a high degree of sulfonation, was shown to be less sensitive to salinity and temperature. This polymer has been recommended for reservoir temperatures up to 95 °C [20,21,23,24].

SUPERPUSHER SAV55: It has a similar backbone to AN132 VHM, containing acrylamide (AM) and AMPS groups as functional groups. Its higher sulfonation degree is one of the main differences between SUPERPUSHER SAV55 and AN132 VHM, which gives this polymer the capability to be stable even to up to 100 °C [21].

THERMOASSOCIATIF: This copolymer is from the family of stimuli-responsive polymers which is capable of associating at specific temperatures (thermoassociative). This polymer is also called thermo-responsive [21] (or thermosensitive [20]) copolymer, which has been observed to show thermo-thickening behavior at high temperatures and in high salinity conditions [20,21]. This polymer can have an economic benefit in a reservoir with harsh conditions because a lower concentration of polymer will be required to attain the desirable viscosity target compared to other polymers. These polymers consist of main chains which are water soluble with blocks or side groups with LCST (lower critical solution temperature) moieties. The backbone of the THERMOASSOCIATIF and AN132 VHM copolymer are alike, but the THERMOASSOCIATIF polymer has been through several structural modifications.

**Figure 3:**
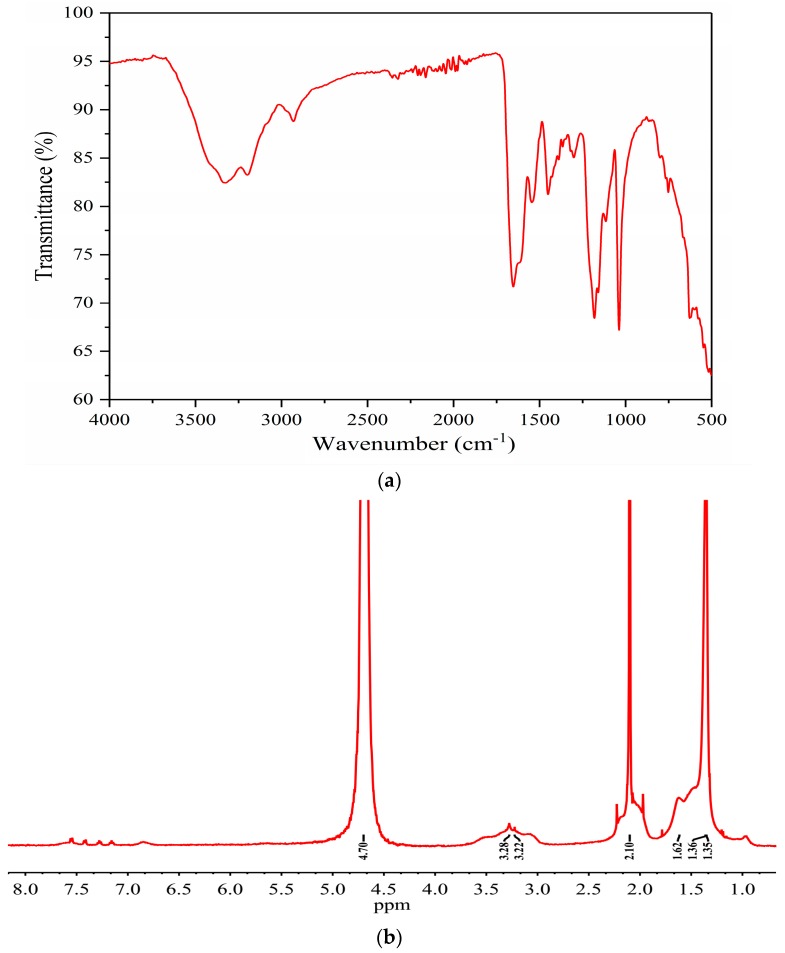
FTIR (**a**) and NMR (**b**) spectra of THERMOASSOCIATIF copolymer.

The FTIR (Fourier transform infrared spectroscopy) and NMR (nuclear magnetic resonance) spectra of the THERMOASSOCIATIF copolymer are presented in Figure 3. FTIR was performed on the polymer powder using a Nicolet™ iS™ 10 FT-IR Spectrometer (Atkinson, NH, USA) and NMR was conducted for the aqueous solution of the polymer and D_2_O using a Bruker 500 NMR spectrometer (Bruker Biospin, Fällanden, Switzerland). In the FTIR spectrum provided in Figure 3a, the C=O stretching vibrations of the –CONH_2_ may possibly be assigned to the peak at 1652 cm^−1^. The N–H stretching vibrations of the –CONH_2_ group and –CH_3_ appeared at the wavenumbers 3325 and 2930 cm^−1^, respectively. Peaks at 1037 and 1181 cm^−1^ may involve the –SO_3_^−^ group [25]. In the NMR spectrum provided in Figure 3b, the hydrogens in the main chain appeared at the peaks between at 1.1–1.7 ppm. The hydrogen atoms of the CH_3_ groups were observed at 2.10 ppm. The hydrogens of the CH_2_ group bonded to SO_3_Na were detected at about 3.2 ppm [26]. The peak at 4.7 ppm is attributed to the hydrogens of the NH and NH_2_ groups and the water in the solvent D_2_O (99.9%) and in the copolymer [27].

Table 1 provides the list of tested products as well as their characteristics. All four types of the copolymers were thankfully provided in powder form by SNF Floerger (ANDREZIEUX Cedex, France).

**Figure 4:**
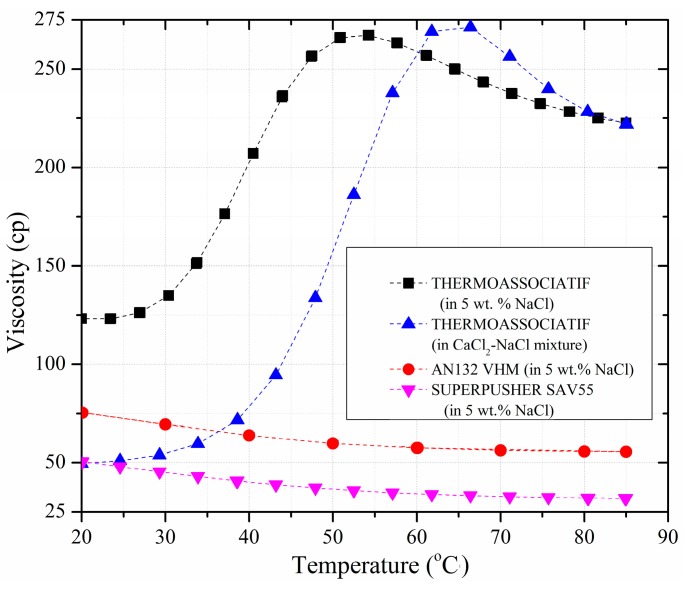
Comparison of rheological profiles of the copolymers at 4500 ppm polymer concentration as a function of temperature.

Figure 4 compares the rheology of the above-mentioned polymers at 4500 ppm polymer concentration as a function of temperature. As evident in the Figure 4, higher temperatures resulted in lower viscosity for standard copolymers (AN132 VHM and SUPERPUSHER SAV55). However, the same test led to unusual results for the thermo-sensitive polymer. As shown in Figure 4, after a certain transition temperature—LCST between 25 and 30 °C for both the 5 wt % NaCl solution and the CaCl_2_-NaCl mixture—the viscosity increased sharply and reached a peak at a specific temperature (55 °C for the 5 wt % NaCl solution and the 65 °C for CaCl_2_-NaCl mixture). At temperatures near the LCST, the tendency of the LCST parts to phase separately is the main reason for the formation of intermolecular aggregates. Due to the aggregation, cross-linking of the polymer chains occurs and therefore the viscosity increases as the temperature is raised. Note that the thermos-thickening behavior of thermos-responsive system is reversible and the same viscosity for 25 °C was obtained when the temperature decreased from 85 to 25 °C. Moreover, the brine composition had a considerable impact on the zone where the transition occurred, as evident in Figure 4 for the 5 wt % NaCl and CaCl_2_-NaCl mixture. This implies that the transition can even be shifted to occur at lower or higher temperatures.

### 2.2. Brine Preparation

The two solvents used in this study are listed in Table 2. It can be noted that both solvents have the same ionic strengths.

### 2.3. Polymer Solution Preparation

To prepare solutions of 4500 ppm polymer concentration, the solvents were added and stirred by a propeller at 700 rpm just before adding polymer powder. The polymer was added into the solvent slowly while the stirrer continued to rotate at a high speed of 700 rpm. This strategy was adopted to avoid the agglomeration of polymer particles, since it has been reported that agglomeration could take place if the polymer does not become wetted immediately [24,28]. 

Aluminum foil was used to cover the beaker so as to minimize the contact with air while mixing continued. After 30 min, the stirrer speed was reduced to 400 rpm and was further stirred for one night to guarantee complete hydration of the polymer and to obtain a homogenous polymer solution. The prepared samples were then put inside 30-mL vials and the lid was closed. For samples in which antioxidant was also added, a silicone glue was applied to make the vials air-tight.

### 2.4. Viscosity Measurement

Polymer solutions, as soon as they were prepared, were put inside an oven set at 80 °C and kept there for various times as per the aging requirements of the test. When a viscosity measurement was to be made, a sample vial was taken out of the oven, cooled rapidly in an ice bath, and then brought for viscosity measurement using a rheometer. Rheological profiles were obtained using a Bohlin Gemini 2 (Malvern Instruments, Malvern, UK) with cone-plate geometry (1°, 4 cm). Polymer solution viscosity was measured at a shear rate interval beginning at 1 s^−1^ and ending at 300 s^−1^. The measurements were repeated in reverse order of shear rates, i.e., from 300 to 1 s^−1^, however, no share rate hysteresis was observed. All measurements were taken after 15 s of rate adjustment.

In EOR polymer flooding projects, the shear rates in the reservoir are higher near the injection well and gradually decrease as the polymer front advances away from the well bore due to radial flow geometry as a result of the increasing cross-sectional area available to the polymer front. In most reservoirs, however, shear rates of 1 to 20 s^−1^ are expected away from the close proximity of the injection well [16]. The higher shear rates near the well are generally not the focus because their range is very small. However, the 1 to 300 s^−1^ range was selected for this study to cover the entire range of polymer advancement from the injection well to the production well or reservoir external boundary.

All viscosity measurements were performed under aerobic conditions and at a temperature of 25 °C. The viscosity variations are more accurate and stability is less hindered by viscometer reading error when tests are performed at low temperatures. However, the low temperature measurements are inadequate for thermoresponsive polymers since they show their thermoresponsive properties only at high temperature [21].

### 2.5. Oxygen-Free Environment

Polymer oxidation could occur rather quickly in the presence of oxygen at elevated temperatures. This short-term mechanism breaks down long molecular chains of polymers, thereby reducing the viscosity [5,6]. 

Fortunately, most reservoirs have a reducing environment, and the produced waters typically contain no detectable dissolved oxygen. With good management of surface facilities (inert gas blanketing, minimizing leaks, and gas stripping where necessary), recycled produced water can be used to prepare EOR solutions that are oxygen free. Oxygen scavengers and antioxidants can also be used as needed to avoid oxygen contamination [12,16,29]. 

To obtain such oxygen-free conditions for laboratory experiments, the preparation of polymer solution inside an anaerobic chamber is one of the ideas proposed [12,20,21]. The addition of antioxidant chemicals into the polymer solution has also been proposed [16,18,30]. 

In this study, a later technique is adopted, i.e., iso-butyl-alcohol (IBA) was added to obtain oxygen-free conditions and thus prevent oxidization of the polymer. This is because the alcohol can be easily oxidized and thereby acts as a sacrificial agent to protect the polymer against oxidization [31,32,33]. Several researchers have used antioxidants, albeit of different kinds of alcohols, but IBA has shown better protection from the harmful effect of oxidization on the reduction of viscosity of polyacrylamide-based polymers [31,32,33].

## 3. Results and Discussion

### 3.1. Aging Time Dependence of Polymer Viscosity at 80 °C in 5 wt % NaCl without Sacrificial Agents

**Figure 5:**
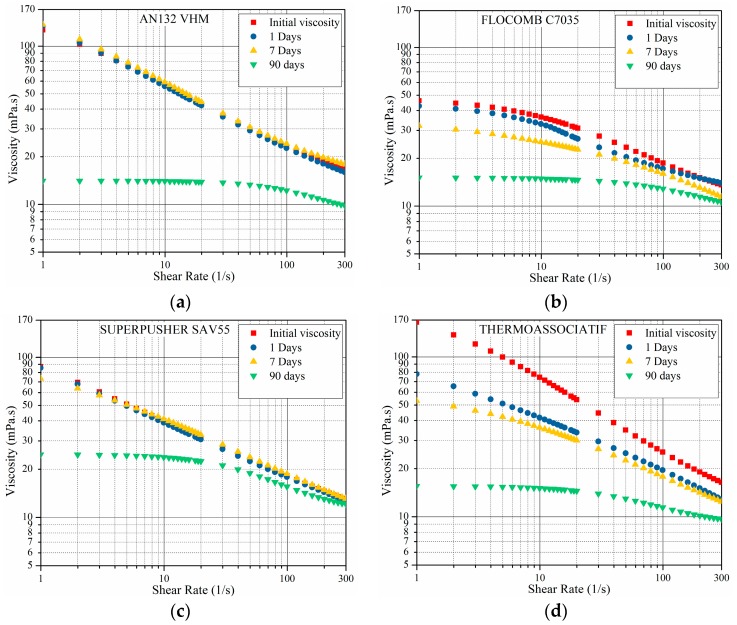
The viscosity as a function of shear rate measured at 25° C for polymers in 5 wt % NaCl (without IBA). Samples varied in aging time, ranging from 0 to 90 days at 80 °C.

The rheological behavior of the four newly-modified sulfonated co-polymers is shown in Figure 5. The samples varied in aging time, ranging from 0 to 90 days at 80 °C, however, the viscosity measurements were performed at 25 °C. The samples were prepared in 5 wt % NaCl alone, thus no divalent ions or antioxidant (IBA) were present. As in the other tests in this part, the prepared samples were then put inside 30-mL vials and the lid was closed. Since there was no IBA, there was no need to make the vials air-tight by applying silicone glue. Figure 5 clearly shows the following:

Viscosity decreased as sample aging time increased for all four polymers. This behavior is expected by virtue of two known phenomena, namely polymer hydrolysis and polymer oxidation. In hydrolysis—a long term process—acrylamide moieties are hydrolyzed to negatively charged acrylate, increasing the intra-chain charge repulsion, thus causing an increase in viscosity [15]. In polymer oxidation, on the other hand, the presence of oxygen forms free radicals, a process which is enhanced at higher temperatures. Though molecular weight reduction was not measured in this study, it is surmised that free radicals might have significantly cleaved the polymer backbone, reducing the molecular weight of the polymer, thereby reducing the viscosity. This phenomenon has been suggested by previous researchers as well [15,16,31]. This chemical degradation of both HPAM and sulfonated co-polymers is significant and quick acting. All in all, the results show that the polymer oxidation effect is much more pronounced than the hydrolysis effect and all studied polymers experience viscosity reduction at high temperatures. In practice, results show that for field application, special treatment should be considered to avoid presenting oxygen in the polymer flooding process. The “AN132 VHM” and “SUPERPUSHER SAV55” withstood aging up to at least seven days (second aging interval) without viscosity degradation, probably due to the high degree of sulfonation, which strengthens the backbone against oxidation [33]. The other two polymers started degrading immediately. For the THERMOASSOCIATIF polymer, structural modifications may be responsible for the chemical instability of the polymer in the presence of oxygen at the elevated temperature.

### 3.2. Effect of Divalent Ions on the Aging Time Dependence of Polymer Viscosity at 80 °C without an Antioxidant

**Figure 6:**
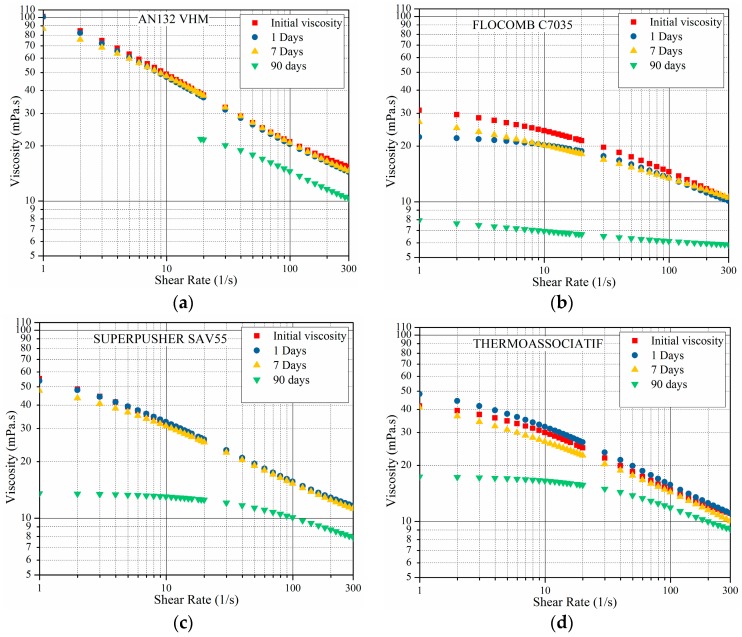
The viscosity as a function of shear rate measured at 25 °C for polymers in CaCl2-NaCl solution of same ionic strength as 5% NaCl and without IBA. Samples varied in aging time, ranging from 0 to 90 days at 80 °C.

In order to investigate the effect of divalent ions on the rheological behavior, the measurements shown in Figure 5 above were repeated under the same conditions but in the presence of a divalent ion. For this set of measurements, all four polymers were prepared in CaCl_2_-NaCl with same ionic strength as the 5 wt % NaCl; the results are shown in Figure 6. 

The addition of Ca^2+^ lowers viscosity for all polymer types as compared to the monovalent ion results shown in Figure 5. This behavior was expected as per published research for the following two reasons: Firstly, cations have more charge screening ability than monovalent ions [20,24,34]. Secondly, divalent ions such as Ca^2+^ can also specifically bind to negative sites on the polymer [4,35,36,37,38]. These bindings can be between COO^−^ groups which formed during polymer hydrolysis on the same polymer molecule (intramolecular). In accordance with this, Flory and Osterheld [37] showed that Ca^2+^ caused a much stronger coil contraction of polyacrylates than the corresponding amount of Na^+^. The contraction was beyond what one would expect from the electrostatic screening effect alone. Intramolecular COO^−^–Ca^2+^–COO^−^ bonds are likely give a decrease in the hydrodynamic volume of the polymer coils, which results in a viscosity reduction.

Figure 6 also shows that viscosity decreased as the sample aging time increased for all four polymers, as was the case in the monovalent ions solutions shown in Figure 5. 

### 3.3. Aging Time Dependence of Polymer Viscosity at 80 °C with an Antioxidant and Only Monovalent Ions

**Figure 7:**
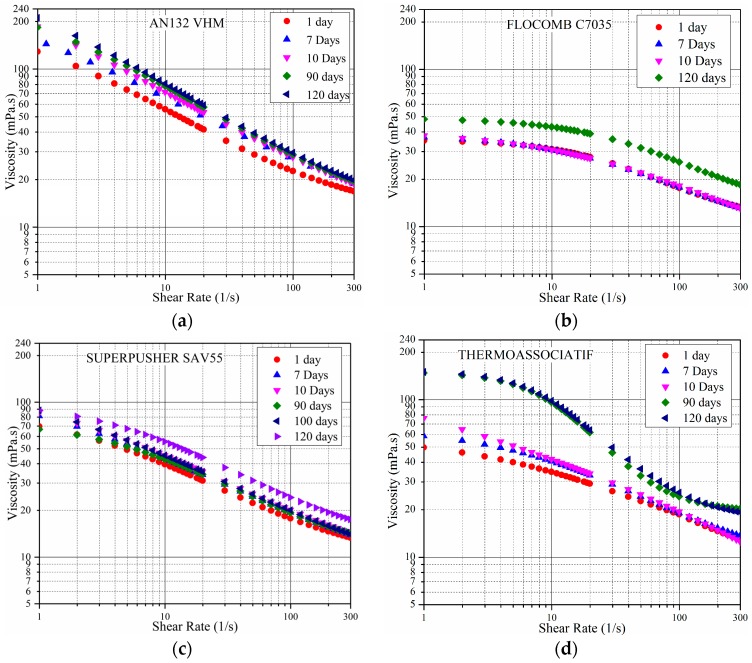
The viscosity as a function of shear rate measured at 25 °C for polymers in 5 wt % NaCl and 3 wt % IBA. Samples varied in aging time, ranging from 1 to 120 days at 80 °C.

As previously described, the presence of oxygen leads to the oxidative degradation of the polyacrylamide polymer, which causes the viscosity to decrease with aging, as shown in Figure 7. However, this effect of dissolved oxygen on polyacrylamide solution viscosity is not significant at low temperatures, thus the polymer solution could be stable for a long time. As the temperature increases, however, in the presence of even small amounts of oxygen, the viscosity decreases with time rather quickly [6]. Addition of an antioxidant to the polymer solution can act as a sacrificial agent, reacting with and consuming the oxygen content completely, thus providing anaerobic conditions.

In order to investigate the rheological behavior in the anaerobic conditions that exist in most reservoirs, the measurements shown in Figure 5 above were repeated under same conditions but in the presence of IBA as a sacrificial agent. For this set of measurements, all four polymers were prepared in 5 wt % NaCl and 3 wt % IBA; the results are shown in Figure 7. Figure 7 shows the following:

In presence of 3 wt % IBA as a sacrificial agent and in the absence of divalent cations, polyacrylamide-based copolymers were stable over 90 days at 80 °C, and in fact showed an increase in viscosity over this aging time. Due to IBA addition, it is assumed that there was no dissolved oxygen. This increase had been reported previously by other researchers [39,40]. This behavior is thought to be the result of the increasing degree of hydrolysis, which generates a higher charge density of anionic functionalities along the polymer backbone at elevated temperatures [4,6,15].

AN132 VHM experienced more hydrolysis than SUPERPUSHER SAV55, which in turn results in higher viscosity increment. It has been reported that the higher the sulfonation degree, the better the resistance to hydrolysis [33,41]. SUPERPUSHER SAV55 has a higher sulfonation degree as compared to the AN132 VHM, which can provide better resistance to hydrolysis and thus better sustain the original viscosity. It is interesting to note here that the hydrolysis of the polyacrylamides mainly depends on the temperature, and is largely independent of the brine composition [5,11].

**Figure 8:**
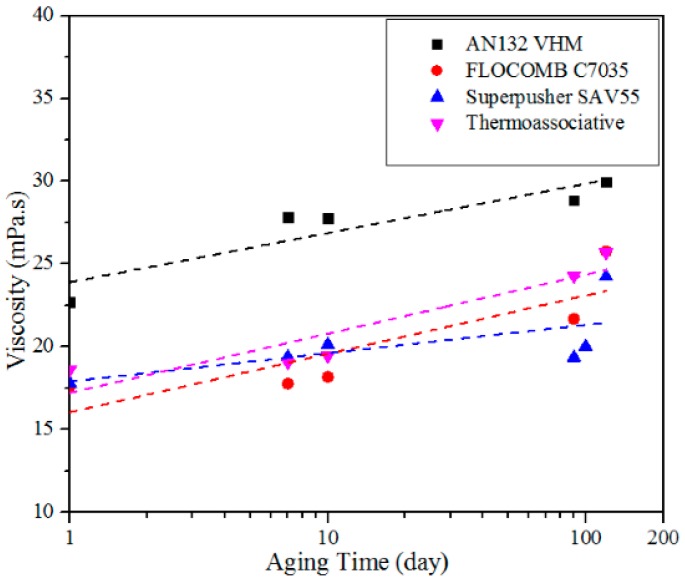
The viscosity as a function of aging time at a fixed shear rate of 100 s−1 measured at 25 °C for polymers in 5 wt % NaCl and 3 wt % IBA. Samples varied in aging time, ranging from 1 to 120 days at 80 °C.

To highlight the behavior further, the rheological behavior of the four polymers was examined at a fixed shear rate of 100 s^−1^ for polymers prepared in 5 wt % NaCl and 3 wt % IBA. The results are shown in Figure 8. No further explanation is provided since this is similar to the measurements in Figure 7, albeit at a fixed shear rate.

### 3.4. Effect of IBA Concentration on the Viscosity

**Figure 9:**
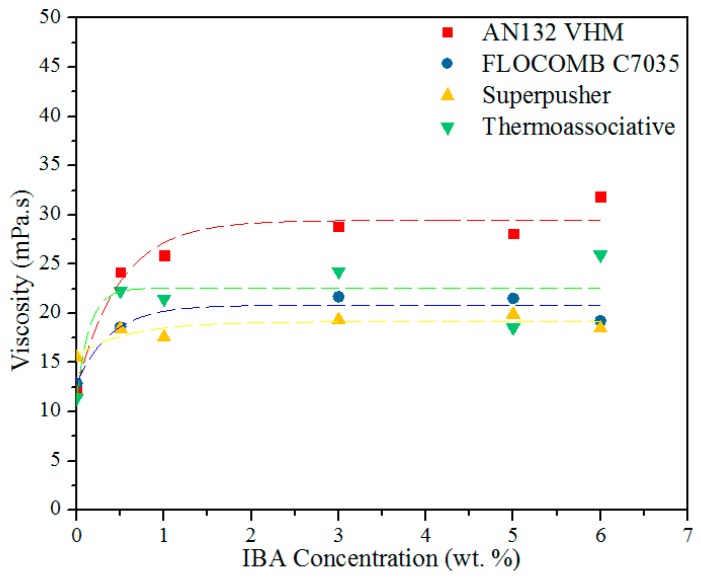
The viscosity of polymers as a function of IBA concentration after 90 days aging time at 80 °C, 5 wt % NaCl, and at 100 s−1.

The effect of IBA concentration on the viscosity behavior of the four newly-modified sulfonated co-polymers is shown in Figure 9. This is important to validate the relevance of our measurements at 3 wt % IBA. Figure 9 shows the following:

The viscosity increased with an increase in IBA concentration up to 3 wt %, after which its effect leveled off, i.e., there was no significant change in viscosity with an increase in IBA concentration higher than 3 wt %. The maximum IBA concentration for this test was 6 wt % because it was thought that all oxygen would be consumed by this concentration, which was in line with other researchers’ observations [32,33].

### 3.5. Effect of Divalent Ions on the Aging Time Dependence of Polymer Viscosity at 80 °C with an Antioxidant

**Figure 10:**
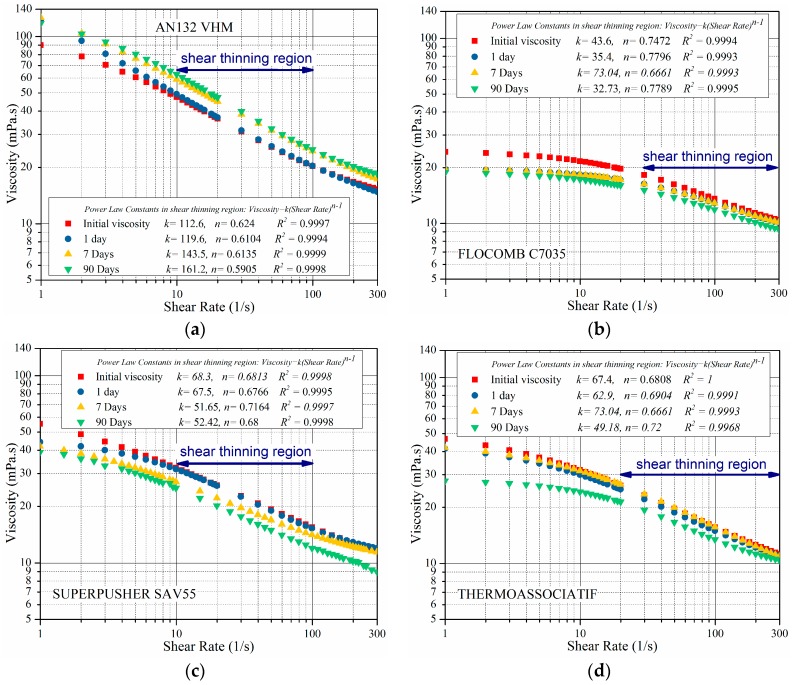
The viscosity as a function of shear rate for polymers aged up to 90 days at 80 °C, and in CaCl2-NaCl including 3 wt % IBA.

In order to investigate the effect of divalent ions on the rheological behavior in the presence of sacrificial agents, the measurements shown in Figure 7 above were repeated under same conditions but in the presence of a divalent ion. For this set of measurements, all four polymers were prepared in CaCl_2_-NaCl with same ionic strength as of 5 wt % NaCl; the results are shown in Figure 10.

Figure 10 shows that for AN132 VHM, when compared with the results in Figure 7, the rate of increasing viscosity was reduced as a result of using CaCl_2_-NaCl in the solution instead of 5 wt % NaCl. This viscosity reduction was likely due to the presence of Ca^2+^ in the solution, which caused coil contraction (reduction in radius of gyration).

Figure 10 also shows that for polymers other than AN132 VHM, the viscosity reduced with increased aging time, in contrast to the polymer viscosity behavior in the 5 wt % NaCl solvent. This is due to the effect of coil contraction and strong binding between divalent ions and the carboxylate group, causing viscosity reduction. The above two effects were powerful enough not only to overcome the viscosity increment caused by polymer hydrolysis, but further to cause a viscosity reduction. This behavior is in line with previous findings [18]. 

However, this reduction is not as significant as the viscosity reduction observed in Figure 5 and Figure 6, which took place in the presence of oxygen. Therefore, it can be concluded that the elimination of even a small amount of dissolved oxygen for high temperature practices is a necessity [6]. 

A comparison between the viscosity profile of AN132 VHM and SUPERPUSHER SAV55 in solutions containing divalent ions shows that AN132 VHM maintained its viscosity in the presence of divalent ions much better than SUPERPUSHER SAV55. It may be due to its higher molecular weight requiring more power for coiling up the polymer macromolecule, thus reducing the viscosity. This observation is in line with research published by many researchers who reduced the salt/hardness sensitivity of PAM by synthetizing high-molecular-weight PAM [18].

Figure 10 also shows the coefficients (*k* and *n*) in the power law model ($viscosity=k{\left(shear\;rate\right)}^{n-1})$. These constants were extracted for the shear thinning regime by using the curve fitting toolbox of MATLAB. It is evident from Figure 10 that polymer viscosity behavior followed power law fit distributions with a negative slope and a high correlation coefficient of *R*^2^ > 0.99.

## 4. Observations and Conclusions

Rheological behaviors and thermal stability were comparatively studied for four types of new copolymers, namely AN132VHM (medium molecular weight, high anionicity and sulfonation degree), FLOCOMB C7035 (high molecular weight, medium anionicity and low sulfonation degree), SUPERPUSHER SAV55 (low molecular weight, high anionicity and sulfonation degree), and THERMOASSOCIATIF (medium molecular weight medium, medium sulfonation degree and anionicity). Each of these copolymer types were mixed in two different solvent, a 5 wt % NaCl and CaCl_2_-NaCl solution of same ionic strength. The observations and conclusions from this study are as follows:

1. In the absence of an antioxidant:
  - All samples maintained their viscosity very well up to seven days, except the THERMOASSOCIATIF polymer which started degrading immediately.
  - After aging for 90 days, all studied polymers, whether prepared in 5 wt % NaCl or CaCl_2_-NaCl, showed severe viscosity reduction due to polymer oxidation.
2. In the presence of isobutyl alcohol (IBA) as an antioxidant:
  - All polymer samples that were prepared using the 5 wt % NaCl solvent showed a gradual increase in viscosity as the aging time increased (up to 90 days of test duration) due to polymer hydrolysis at high temperatures.
  - For the samples that were prepared with 5 wt % NaCl as the solvent, viscosity increased after 90 days of aging (which can be thought of as increased thermal stability), as the IBA amount was increased up to 3 wt %, after which the addition of more IBA did not have a significant effect on viscosity.
  - For the samples which were prepared in the CaCl_2_-NaCl solvent, all polymers except AN132 VHM showed a viscosity reduction after 90 days of aging due to the strong ability of cations in charge screening and binding to negative sites on the polymer.
  - AN132 VHM, on the other hand, showed a viscosity increase after 90 days of aging, which implies that the viscosity increase rate due to polymer hydrolysis was higher than the viscosity reduction rate due to the presence of divalent ions in the solution.

## Figures and Tables

**Table 1:** Polymer characteristics.

Polymer Product (Trade Name)	Molecular Weight (Million Daltons)	Sulfonation Degree (mol %)	Anionicity
FLOCOMB C7035	Very High (>18)	low	Medium
AN132 VHM	Medium (9–11 ^1^)	32	High
SUPERPUSHER SAV 55	Low (5–7 ^1^)	high	High
THERMOASSOCIATIF	Medium (<12)	medium	Medium

^1^ Data has been provided by SNF Floerger, except the numerical value (indicated by 1) which was extracted from SPE-177073-MS [16].

**Table 2:** Brine composition.

Brine Name	NaCl (g/kg of Solvent)	CaCl_2_ (g/kg of Solvent)	Deionized Water (g/kg of Solvent)
5 wt % NaCl ^1^	50	0	950
CaCl_2_-NaCl ^1^	20	19	961

^1^ Note: Sodium chloride and calcium chloride were provided by J.T. Baker (Phillipsburg, NJ, USA).
